# Decreased expression of *SCARA5* predicts a poor prognosis in melanoma using bioinformatics analysis

**DOI:** 10.3389/fonc.2023.1015358

**Published:** 2023-03-24

**Authors:** Qinggan Ni, Xia Li, Hua Huang, Zili Ge

**Affiliations:** ^1^ Department of Oral and Maxillofacial Surgery, The First Affiliated Hospital of Soochow University, Suzhou, Jiangsu, China; ^2^ Department of Burns and Plastic Surgery, Yancheng Clinical College of Xuzhou Medical University, The First People’s Hospital of Yancheng, Yancheng, China; ^3^ Department of General Medicine, Yancheng Third People’s Hospital, The Sixth Affiliated Hospital of Nantong University, Yancheng, Jiangsu, China; ^4^ Department of Pathology, Affiliated Hospital of Nantong University, Nantong, Jiangsu, China

**Keywords:** *SCARA5*, melanoma, tumor-infiltrating, immunohistochemistry, bioinformatics analysis

## Abstract

**Background:**

It has been established that the scavenger receptor class A member 5 (*SCARA5*) functions as a tumor suppressor gene in various cancer types. To our knowledge, no comprehensive study has hitherto investigated the expression and function of *SCARA5* in melanoma. This study aimed to determine the association between *SCARA5* and melanoma.

**Methods:**

Analysis of *SCARA5* mRNA expression was performed using The Cancer Genome Atlas (TCGA) data sets. To evaluate the clinical significance of *SCARA5*, the clinical data of 93 patients with melanoma were collected. The role of *SCARA5* expression in prognosis was also analyzed. In this study, survival was evaluated by Kaplan–Meier analysis and compared using the log-rank test. Univariate and multivariate Cox proportional hazard regression analyses were used to identify independent predictors. The Kyoto Encyclopedia of Genes and Genomes, Gene Ontology, and gene set enrichment analysis (GSEA) were used to perform gene set functional annotations. Protein–protein interaction (PPI) networks were constructed to illustrate gene–gene interactions. The Tumor IMmune Estimation Resource (TIMER) database was used to explore the association between *SCARA5* and immune infiltration levels.

**Results:**

The results showed that the *SCARA5* mRNA expression in melanoma was significantly lower than in adjacent normal skin tissue (*p* < 0.001). Moreover, decreased expression of *SCARA5* in melanoma correlated with the tumor, node, and metastasis (TNM) stage and recurrence (*p* < 0.05). The overall survival (OS) was significantly higher in melanoma with high *SCARA5* expression compared with low *SCARA5* expression (*p* < 0.001). During univariate analysis, *SCARA5* expression, tumor (T) stage, node (N) stage, metastasis (M) stage, and recurrence correlated with OS (*p* < 0.05). Further multivariate Cox regression analysis showed that *SCARA5* expression (*p* = 0.012) could be an independent prognostic factor for OS in cutaneous malignant melanoma. GSEA analysis showed that *SCARA5* was significantly enriched in various pathways, such as response to developmental biology and response to antimicrobial peptides. Correlation analysis showed a positive correlation with CD8+ T cells, CD4+ T cells, macrophages, neutrophils, and dendritic cells (*p* < 0.05), and a negative correlation with tumor purity (*p* < 0.05)

**Conclusion:**

*SCARA5* has significant potential as a prognostic biomarker and as a promising therapeutic target in melanoma. Furthermore, *SCARA5* expression in melanoma is related to the level of immune infiltration.

## 1 Introduction

Malignant melanoma (MM) is a highly malignant tumor originating from epidermal melanocytes that has the characteristics of early metastasis, a high degree of malignancy, rapid development, poor prognosis, and high mortality (1). Over the past 30 years, the incidence of melanoma in the world has risen rapidly, and diagnoses tend to occur in younger people (2, 3). Although malignant melanoma accounts for less than 5% of total skin cancer incidence, it has an extremely high mortality rate, accounting for approximately 75% of all skin cancer mortality (4). U.S. researchers predicted 100,350 new melanoma diagnoses by 2020, with an estimated 6,850 deaths from the disease (5). At present, the pathogenesis of malignant melanoma remains unclear. The treatment approach for malignant melanoma is mainly based on local surgical resection combined with systemic radiotherapy, chemotherapy, immunotherapy, and tumor-targeted gene therapy (6). However, the overall treatment effect is not ideal, owing to the risk of early metastasis in malignant melanoma and the poor sensitivity of radiotherapy and chemotherapy (7, 8). With the rapid development of modern biomedicine, the application of new effective treatment methods such as biomedical approaches and tumor-targeted therapy has broadened the therapeutic landscape for treating malignant melanoma. Therefore, effective molecular markers and biological therapeutic targets can play an important role in diagnosing and treating malignant melanoma (9).

Scavenger receptor class A member 5 (*SCARA5*) is a member of the scavenger receptor (SR) family. The full-length gene is 3.644kb, encoding 495 amino acids and located on chromosome 8 (10). *SCARA5* is a type II transmembrane glycoprotein that binds to a variety of anionic ligands including low-density lipoprotein, serum ferritin, polynucleotides, bacterial metabolites, and modified extracellular matrix proteins. *SCARA5* is a kind of tumor suppressor gene, and its expression is downregulated in many kinds of tumor cells and tissues. Upregulation of *SCARA5* can significantly inhibit the proliferation, cloning, invasion, and migration of the tumor cell (11–16). In addition, *SCARA5* is involved in iron metabolism and plays an important role in autoimmune diseases (17, 18). Huang et al. found that overexpression of *SCARA5* in liver cancer cells can inhibit tumorigenicity, cell invasion, and metastasis (11). Furthermore, *SCARA5* inhibits breast cancer cell proliferation, colony formation, invasion, and migration by inhibiting the phosphorylation of *AKT, STAT3*, and *ERK1/2* and also induces breast cancer cell apoptosis (19). Therefore, the application of *SCARA5* has significant potential as a tumor suppressor.

No study has hitherto reported the effect of *SCARA5* on malignant melanoma. Accordingly, our current study focused on the association of *SCARA5* with prognosis in cutaneous malignant melanoma. We studied and analyzed three microarray data sets from the Gene Expression Omnibus (GEO) containing expression data from melanoma cancer tissue and adjacent normal skin tissue. Differentially expressed genes (DEGs) were identified by Gene Expression Omnibus 2 Recovery (GEO2R), and protein–protein interaction (PPI) networks were subsequently constructed to identify highly connected hub genes. Then, pathway analysis was performed by Gene Ontology (GO), gene set enrichment analysis (GSEA), and the Kyoto Encyclopedia of Genes and Genomes (KEGG). The relationship between *SCARA5* expression and tumor-infiltrating immune cells was analyzed using the Tumor IMmune Estimation Resource (TIMER). We found that high *SCARA5* expression correlated with longer overall survival (OS) in melanoma patients, and *SCARA5* was an independent prognostic factor for OS in melanoma patients. This study suggests that *SCARA5* may serve as a therapeutic target and prognostic indicator for cutaneous melanoma.

## 2 Materials and methods

### 2.1 Databases

The data analyzed in this study were downloaded from the GEO database (http://www.ncbi.nlm.nih.gov/geo) (20), a global gene expression database created by NCBI that contains high-throughput gene expression from research institutions’ gene expression data. We selected three RNA arrays, GSE7553 (21), GSE15605 (22), and GSE100050 (23), as data sets from the GEO database GLP570 platform [(HG-U133_Plus_2) Affymetrix Human Genome U133 Plus 2.0 Array]. The GSE7553 data set consisted of 87 samples (including 82 tumor samples and five normal skin tissue samples), the GSE15605 data set consisted of 74 samples (including 46 primary melanoma, 12 metastatic lesions, and 16 normal skin samples for full genome expression profiling), and there were 12 samples in the GSE100050 data set (including six tumor samples and six normal skin tissues). The number of patients in The Cancer Genome Atlas (TCGA)-Genotype-Tissue Expression (GTEx)-Skin cutaneous melanoma (SKCM) data set was 1,282, comprising normal GTEx (*n* = 812), TCGA para-cancer (*n* = 1), and TCGA tumors (*n* = 469).

### 2.2 Identification of DEGs

Differentially expressed genes between cutaneous malignant melanoma cancer tissue samples and non-cancer samples were screened using the GEO2R tool (http://www.ncbi.nlm.nih.gov/geo/geo2r). GEO2R is an interactive web tool that can compare two or more GEO data sets. To identify DEGs, we applied adjusted (adj.) *p*-values and thresholds to the Benjamini and Hochberg false discovery rates to balance the limitations of finding (statistically) significant genes and false positives. Probe sets that lacked corresponding gene symbols were eliminated, and genes that exhibited multiple probe sets were eliminated. The criteria for significant DEGs included a log-fold change (FC) <1 and adj. *p <*0.01.

### 2.3 KEGG/GO and GSEA analyses of the DEGs

Driver and Vehicle Information Database (DAVID) (http://david.ncifcrf.gov) (version 6.7) (24), an online bioinformatics database with comprehensive analysis tools, was used to conduct KEGG/GO and GSEA analyses of the DEGs. The biological information was extracted by conducting functional annotation of genes and proteins. KEGG is a database that can be used to better understand the biological functions of DEGs (25). GO analysis was used to gain biological insights into the functional role of genes. GSEA enrichment results were visualized using the ggplot2 package.

### 2.4 PPI

Interactions among differential genes and PPI network predictions in this study were performed using the Search Tool for the Retrieval of Interacting Genes/Proteins (STRING; http://string-db.org) (26) online database. The functions of and interactions between proteins were further analyzed by predicting related PPI networks, which can be used to elucidate the pathogenesis of various diseases.

We constructed a PPI network of related DEGs using the online STRING database, and only protein interactions with a composite score of >0.4 were considered statistically significant. The open-source bioinformatics software Cytoscape (version 3.4.0) was used to visualize the molecular interaction network mapping (27). It has been established that the Molecular Complex Detection (MCODE v1.4.2) plug-in from Cytoscape can cluster a given network based on the topology to find densely connected regions (28). Cytoscape was used to draw the PPI network and MCODE to identify the most important modules in the network. The selection criteria were MCODE score >5, degree cutoff = 2, node score cutoff = 0.2, max depth = 100, and k-score = 2. Finally, KEGG and GO analyses were performed on the genes using DAVID.

### 2.5 SKCM patient specimens

To verify *SCARA5* expression in human skin cutaneous melanoma (SKCM), tissue samples from 93 SKCM patients who had not received chemotherapy or radiotherapy were harvested in the Department of Pathology, Nantong University Affiliated Hospital, including paired adjacent non-tumor and tumor SKCM specimens. Fresh samples of resected SKCM tumor tissue and adjacent non-tumor tissue were harvested and stored in liquid nitrogen. Two professional pathologists independently confirmed the tumor grade and histological type of all tissue samples. All patients with cutaneous malignant melanoma provided informed consent, and this study was approved by the Human Research Ethics Committee of the Affiliated Hospital of Nantong University (Nantong, China).

### 2.6 Immunohistochemical staining and assessment of *SCARA5* expression

We used the tissue microarray (TMA) system (Quick-Ray, UT06; UNITMA, Seoul, Korea) of the Department of Clinical Pathology, Affiliated Hospital of Nantong University, based on immunohistochemical staining to assess the expression of *SCARA5* in SKCM. Pathological biopsies of core tissue approximately 2 mm in diameter were obtained from individual paraffin-embedded sections and sequentially arranged in recipient paraffin blocks. TMA blocks were sectioned with a microtome to obtain 4 μm-thick sections placed on glass slides. Hematoxylin and eosin staining was used for quality control in the TMA analysis. Tissue sections were fractionally deparaffinized and rehydrated in graded concentrations of ethanol. Antigen retrieval was performed by boiling the sections in an ethylenediaminetetraacetic acid (EDTA) buffer (pH 6.0) in a pressure cooker for 3 min. Endogenous peroxidase activity was then quenched with 3% hydrogen peroxide for 30 minutes. Sections were then incubated with a *SCARA5*-specific polyclonal antibody (1:50 dilution; Abcam) overnight at 4°C, followed by the biotinylated anti-rabbit secondary antibody for 30 minutes at 37°C. The slides were then treated with a horseradish peroxidase solution and 3,3-diaminobenzidine chromogen, followed by counterstaining with hematoxylin. Tumor and non-tumor tissues were examined for *SCARA5* staining in a blinded fashion. Three fields of view were selected to examine the proportion of positive cells and the intensity of cell staining. Immunohistochemical staining was assessed based on the immunoreactivity score (IRS), assessed by the staining intensity and the proportion of positive cells. Intensity scores were as follows: 0 (negative), 1 (weakly positive), 2 (moderately positive), and 3 (strongly positive). Quantitative scores for the proportion of *SCARA5*-positive cells were recorded according to four categories: 1 (0%–25%), 2 (26%–50%), 3 (51%–75%), and 4 (76%–100%). The IRS (product of intensity score and numerical score) ranged from 0 to 12: an IRS of 0–3 and 4–12 represented low and high *SCARA5* expression, respectively.

### 2.7 Statistical analysis

Statistical analysis was performed using Statistical Product and Service Solutions (SPSS) 20.0 and GraphPad Prism 8.0 software. Differences between the two groups were analyzed by a two-tailed Student’s *t*-test, and quantitative data were presented as mean ± SD. Categorical data were analyzed using a Chi-squared test. A *p*-value of <0.05 was considered statistically significant.

## 3 Results

### 3.1 SKCM-associated DEGs

**Figure 1:**
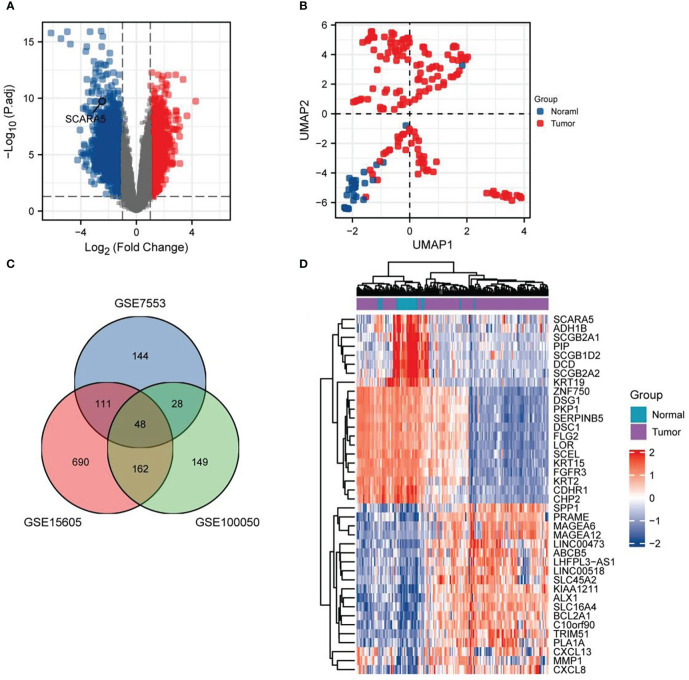
Difference analysis. Volcano plots were used to select differential genes with a threshold oflog FC≥ 1 and a *p*-value ≤ 0.05. Firstly, the differentially expressed gene (DEG) is analyzed by the “Limma” software package, the classic volcano diagram is presented in the form of a volcano diagram, and the location of scavenger receptor class A member 5 (*SCARA5*) is marked **(A)**. Two groups of samples are separated, indicating that the differences between groups is significant, and the subsequent difference analysis is meaningful **(B)**. There are 48 DEGs in the three data sets, comprising nine downregulated and 39 upregulated genes **(C)**. Using the differential expression analysis tool DESeq2, we analyzed the top 20 upregulated and downregulated genes in both cancer tissues and adjacent normal tissues **(D)**.

Data sets GSE7553, GSE15605, and GSE100050 were downloaded from the GEO database through the GEO query package, and the probes corresponding to multiple molecules were removed (29–31). Only the probe with the largest signal value was retained when encountering probes corresponding to the same molecule. After filtering the data, we used the ComBat function of the sva package to eliminate the inter-batch difference. The 173 samples from the three data sets were divided into two groups, comprising 146 samples in the tumor group and 27 samples in the normal group. Volcano plots were used to visualize the significant differential genes with a threshold of |log FC| ≥1 and a *p*-value ≤0.05. First, the DEGs were analyzed by the “Limma” software package and visualized in a volcano plot where *SCARA5* was marked (**Figure 1A**). The two groups of samples were clearly separated, indicating significant differences between both groups (**Figure 1B**). A total of 48 DEGs were identified in the three data sets, consisting of nine downregulated and 39 upregulated genes (**Figure 1C**).

### 3.2 KEGG, GO, and GSEA enrichment analyses

**Figure 2:**
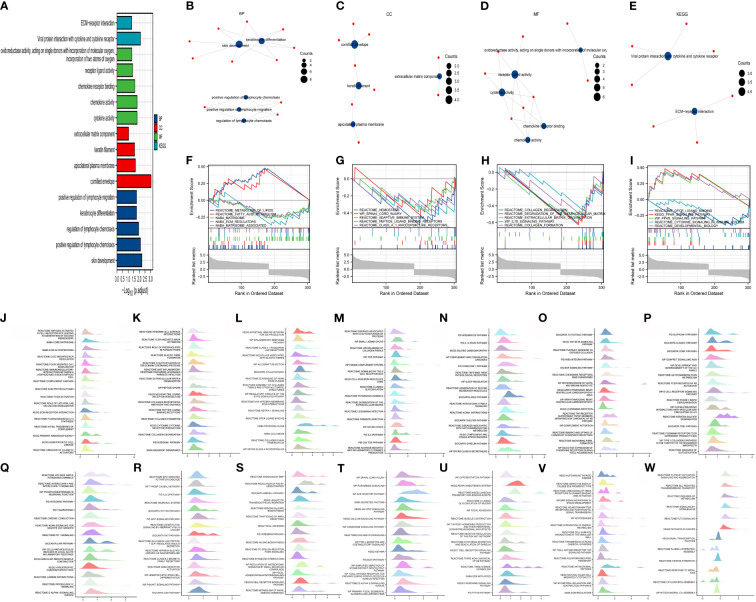
Perform enrichment analysis on 48 differentially expressed genes using the “Cluster Profiler” R package. Under the conditions of adjusted (adj.) *p* ≤ 0.05 and *q*-value ≤ 0.2, there are five biological processes (BPs), four CCs, five molecular functions (MFs), and two Kyoto Encyclopedia of Genes and Genomes (KEGG) pathways **(A)**. The BPs are positive regulation of lymphocyte migration, keratinocyte differentiation, regulation of lymphocyte chemotaxis, positive regulation of lymphocyte chemotaxis, and skin development **(B)**. The CCs are extracellular matrix component, keratin filament, apicolateral plasma membrane, and cornified envelope **(C)**. The MFs are oxidoreductase activity, acting on single donors with incorporation of molecular oxygen, incorporation of two atoms of oxygen, cytokine activity, chemokine activity, chemokine receptor binding, and receptor ligand activity **(D)**. The two KEGG pathways are viral protein interaction with cytokine and cytokine receptor, and ECM–receptor interaction **(E)**. The ggplot2 package was used for gene set enrichment analysis (GSEA). The top 20 pathways mainly enriched by the differentially expressed genes. **(F–I)**, and “ggplot2” was used to set the threshold for significant enrichment as follows: false discovery rate (FDR) < 0.25 and adjust. *p* < 0.05 GSEA gene sets are listed in the form of mountain plots **(J–W)**.

The R package “cluster Profiler” was used for the enrichment analysis of the 48 DEGs. Using the screening criteria adj. *p ≤*0.05 and *q*-value ≤0.2, significantly enriched biological processes (BPs, *n* = 5), cellular components (CCs, *n* = 4), molecular functions (MFs, *n* = 5), and KEGG pathways (*n* = 2) were identified (**Figure 2A**). Significantly enriched BPs comprised positive regulation of lymphocyte migration, keratinocyte differentiation, regulation of lymphocyte chemotaxis, positive regulation of lymphocyte chemotaxis, and skin development (**Figure 2B**). Moreover, the DEGs were significantly enriched in CCs, including the extracellular matrix component, keratin filament, apicolateral plasma membrane, and cornified envelope (**Figure 2C**). In terms of MF, oxidoreductase activity, acting on single donors with incorporation of molecular oxygen, incorporation of two atoms of oxygen, cytokine activity, chemokine activity, chemokine receptor binding, and receptor–ligand activity were enriched (**Figure 2D**). Finally, the DEGs were involved in two KEGG signaling pathways: viral protein interaction with cytokine and cytokine receptor, and ECM-receptor interaction (**Figure 2E**). Subsequently, the ggplot2 package was used to visualize the GSEA enrichment results (**Figures 2F–I**) using the threshold false discovery rate (FDR) of <0.25 and adjusted *p*-value of <0.05. The enrichment scores of the GSEA gene sets can be visualized in the enrichment plots (**Figures 2J–W**).

### 3.3 The PPI network and module analysis

**Figure 3:**
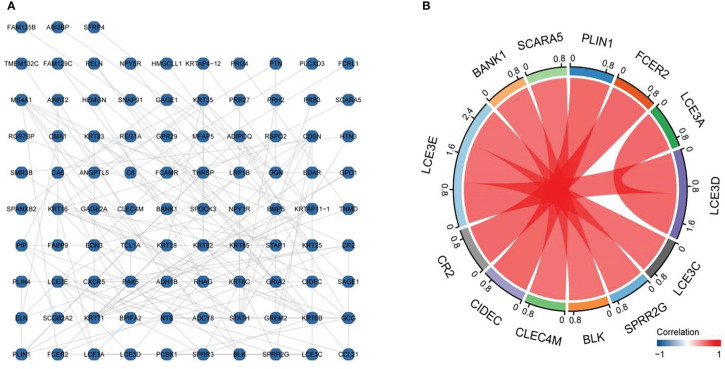
Protein–protein interaction (PPI) network diagram and important modules. The established differentially expressed gene (DEG)-related PPI networks were visualized using the igraph and ggraph packages **(A)**, with the most important module shown in **(B)**.

The established DEG-related PPI networks were visualized using the R packages igraph and ggraph (32) (**Figure 3A**), with the most important module shown in **Figure 3B**. Genes with high correlation coefficients were significantly enriched in skin development, positive regulation of lymphocyte chemotaxis, regulation of lymphocyte chemotaxis, keratinocyte differentiation, positive regulation of lymphocyte migration, cornified envelope, apicolateral plasma membrane, keratin filament, extracellular matrix component, cytokine activity, chemokine activity, chemokine receptor binding, receptor–ligand activity, oxidoreductase activity, acting on single donors with incorporation of molecular oxygen, incorporation of two atoms of oxygen, viral protein interaction with cytokine and cytokine receptor, and ECM–receptor interaction (**Table 1**).

**Table 1:** Gene Ontology (GO)/Kyoto Encyclopedia of Genes and Genomes (KEGG) enrichment analysis of 48 overlapping genes.

Ontology	ID	Description	Gene ratio	Bg ratio	*p*-value	adjust. *p*-value	*q*-value
BP	GO:0043588	Skin development	8/45	419/18670	6.27e-06	0.006	0.005
BP	GO:0140131	Positive regulation of lymphocyte chemotaxis	3/45	21/18670	1.69e-05	0.008	0.007
BP	GO:1901623	Regulation of lymphocyte chemotaxis	3/45	27/18670	3.68e-05	0.012	0.010
BP	GO:0030216	Keratinocyte differentiation	6/45	305/18670	8.62e-05	0.018	0.015
BP	GO:2000403	Positive regulation of lymphocyte migration	3/45	37/18670	9.60e-05	0.018	0.015
CC	GO:0001533	Cornified envelope	4/46	65/19717	1.58e-05	9.02e-04	6.66e-04
CC	GO:0016327	Apicolateral plasma membrane	2/46	18/19717	7.96e-04	0.023	0.017
CC	GO:0045095	Keratin filament	3/46	95/19717	0.001	0.027	0.020
CC	GO:0044420	Extracellular matrix component	2/46	51/19717	0.006	0.090	0.066
MF	GO:0005125	Cytokine activity	5/45	220/17697	2.31e-04	0.016	0.012
MF	GO:0008009	Chemokine activity	3/45	49/17697	2.61e-04	0.016	0.012
MF	GO:0042379	Chemokine receptor binding	3/45	66/17697	6.29e-04	0.025	0.020
MF	GO:0048018	Receptor–ligand activity	6/45	482/17697	0.001	0.040	0.031
MF	GO:0016702	Oxidoreductase activity, acting on single donors with incorporation of molecular oxygen, incorporation of two atoms of oxygen	2/45	27/17697	0.002	0.046	0.036
KEGG	hsa04061	Viral protein interaction with cytokine and cytokine receptor	4/22	100/8076	1.36e-04	0.008	0.007
KEGG	hsa04512	ECM–receptor interaction	3/22	88/8076	0.002	0.048	0.045

CC, Cellular component; BP, Biological process; MF, Molecular function; KEGG, Kyoto Encyclopedia of Genes and Genomes TAM,Tumor Associated Macrophage; ACC, Adrenocortical carcinoma; BLCA, Bladder Urothelial Carcinoma; CESC, Cervical squamous cell carcinoma and endocervical adenocarcinoma; CHOL, Cholangiocarcinoma; COAD, Colon adenocarcinoma; COADREAD, Colon adenocarcinoma/Rectum adenocarcinoma Esophageal carcinoma; DLBC, Lymphoid Neoplasm Diffuse Large B-cell Lymphoma; ESCA, Esophageal carcinoma; GBM ,Glioblastoma multiforme; GBMLGG, Glioma; HNSC, Head and Neck squamous cell carcinoma; KICH, Kidney Chromophobe; KIRC, Kidney renal clear cell carcinoma; KIRP, Kidney renal papillary cell carcinoma; LAML, Acute Myeloid Leukemia; LGG, Brain Lower Grade Glioma; LIHC, Liver hepatocellular carcinoma; LUAD, Lung adenocarcinoma; LUSC, Lung squamous cell carcinoma; MESO, Mesothelioma; OV, Ovarian serous cystadenocarcinoma; PAAD, Pancreatic adenocarcinoma; PCPG, Pheochromocytoma and Paraganglioma; PRAD, Prostate adenocarcinoma; READ, Rectum adenocarcinoma; SARC, Sarcoma; SKCM, Skin Cutaneous Melanoma; STAD, Stomach adenocarcinoma; STES, Stomach and Esophageal carcinoma; TGCT, Testicular Germ Cell Tumors; THCA, Thyroid carcinoma; THYM, Thymoma; UCEC, Uterine Corpus Endometrial Carcinoma; UCS, Uterine Carcinosarcoma; UVM, Uveal Melanoma.

### 3.4 Pan-cancer analysis of *SCARA5* expression

**Figure 4:**
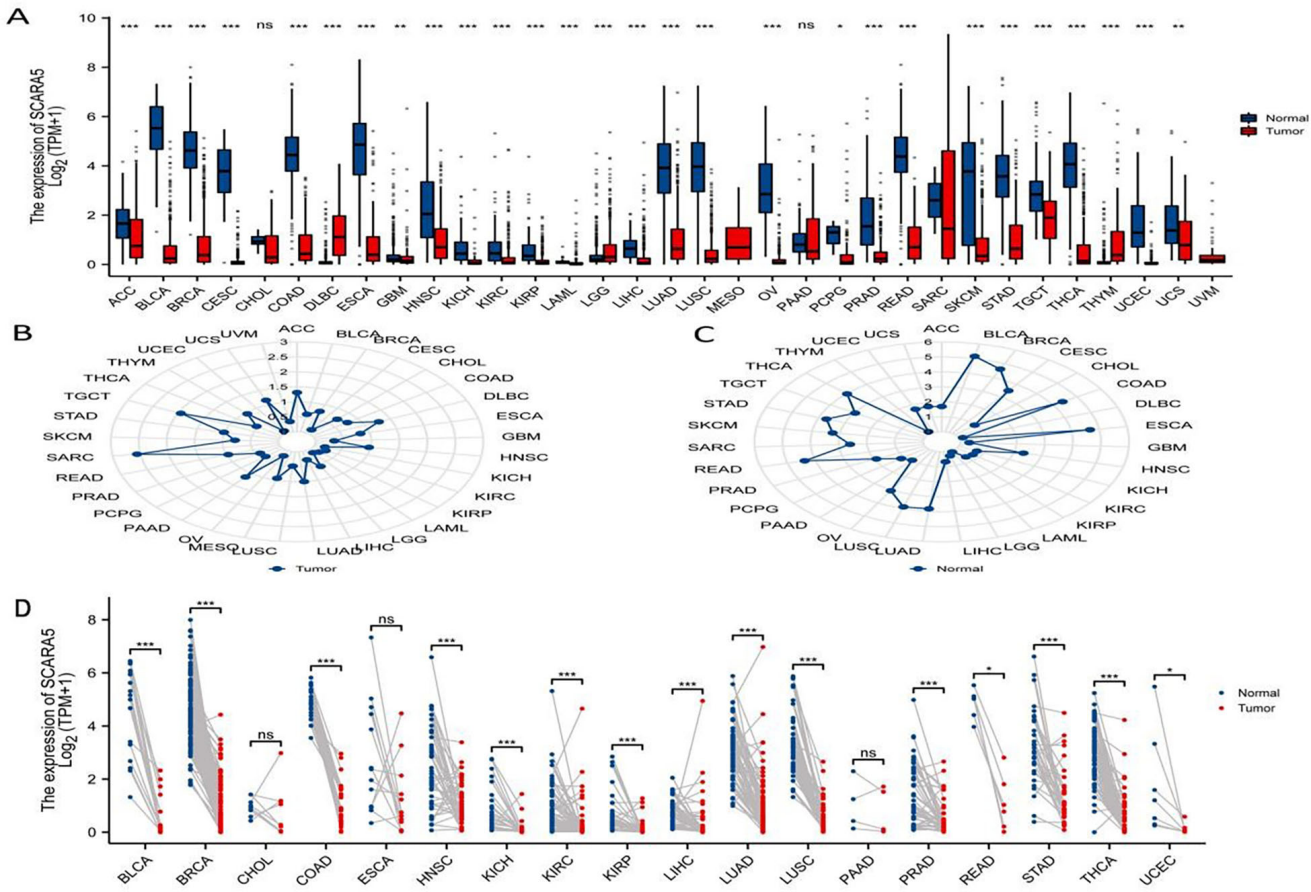
Pan-cancer analysis of scavenger receptor class A member 5 (*SCARA5*). The “ggplot2” package was used to analyze the differential expression of *SCARA5* in pan-cancer cancer tissues and corresponding adjacent tissues: ns, *p* ≥ 0.05; *, *p* ≤ 0.05; **, *p* ≤ 0.01; ***, *p* ≤ 0.001 **(A)**.Radar visualization with “ggradar” and “ggplot2” packages was used to analyze the expression of *SCARA5* in pan-cancer cancer tissues **(B)** and the expression of *SCARA5* in normal tissues adjacent to pan-cancerous tumors **(C)**. The RNA sequencing (RNAseq) data in transcripts per million reads (TPM) format was analyzed and compared after log2 transformation and analysis of differences in *SCARA5* in paired samples. Significance: ns, *p* ≥ 0.05; *, *p* ≤ 0.05; **, *p* ≤ 0.01; ***, *p* ≤ 0.001 **(D)**.

Using the differential expression analysis tool DESeq2, we analyzed the top 20 upregulated and downregulated genes in both cancer tissues and adjacent normal tissues (**Figure 1D**). In addition, the R package “ggplot2” was used to analyze the differential expression of *SCARA5* in pan-cancer tissues and corresponding adjacent tissues (1), and the following groups were retained: *ACC, BLCA, BRCA, CESC, CHOL, COAD, DLBC, ESCA, GBM, HNSC, KICH, KIRC, KIRP, LAML, LGG, LIHC, LUAD, LUSC, MESO, OV, PAAD, PCPG, PRAD, READ, SARC, SKCM, STAD, TGCT, THCA, THYM, UCEC, UCS,* and *UVM*. The following significance markers were used: ns, *p* ≥0.05; *, *p ≤*0.05; **, *p ≤*0.01; ***, *p ≤*0.001 (**Figure 4A**). Radar charts were generated using the “ggradar” and “ggplot2” packages to analyze the expression of *SCARA5* in pan-cancer tissues (**Figure 4B**) and normal tissues adjacent to pan-cancerous tumors (**Figure 4C**). The RNA sequencing (RNA-seq) data, given in transcripts per million reads (TPM), were analyzed and compared after log2 transformation, and the following groups were retained for analysis of differences in *SCARA5* in paired samples (**Figure 4D**): BLCA, BRCA, CHOL, COAD, ESCA, HNSC, KICH, KIRC, KIRP, LIHC, LUAD, LUSC, PAAD, PRAD, READ, STAD, THCA, and UCEC.

### 3.5 Clinical value of *SCARA5* in prognosis

**Figure 5:**
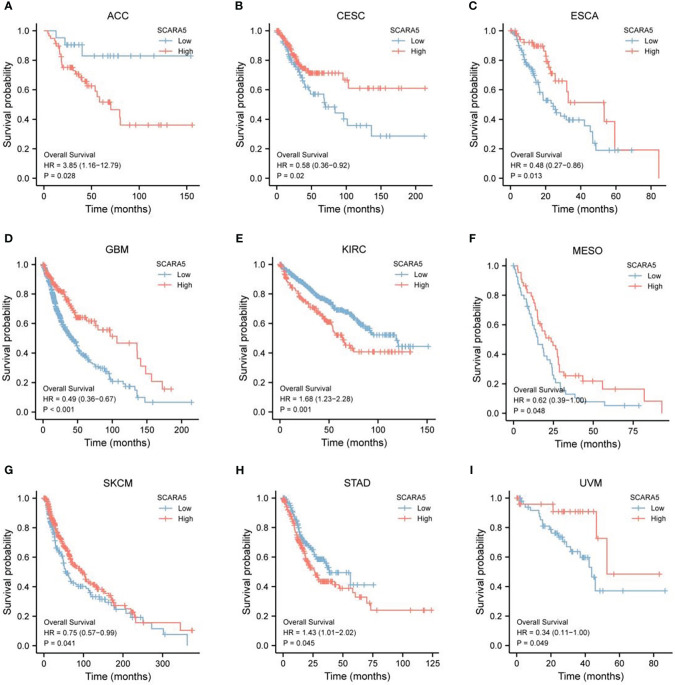
The Cancer Genome Atlas (TCGA)database https://portal.gdc.cancer.gov/) pan-cancer survival data for scavenger receptor class A member 5 (*SCARA5*) expression was divided into a high expression group (50–100) and a low expression group (0–50). Log-rank test statistical method analysis, “survminer” was used for visualization, and it was found that the difference in survival time between *SCARA5* groups in ACC, CESC, ESCA, GBM, KIRC, Skin cutaneous melanoma (SKCM), MESO, STAD, and UVM tumors was statistically significant **(A–I)**.

The pan-cancer survival data from the TCGA database were divided into high-*SCARA5* expression (50%–100%) and low-*SCARA5* expression (0%–50%) groups (https://portal.gdc.cancer.gov/). The log-rank test showed that the difference in survival between *SCARA5* groups in ACC, CESC, ESCA, GBM, KIRC, SKCM, MESO, STAD, and UVM tumors was statistically significant (**Figures 5A–I**).

### 3.6 Relationship between *SCARA5* expression and tumor-infiltrating immune cells

**Figure 6:**
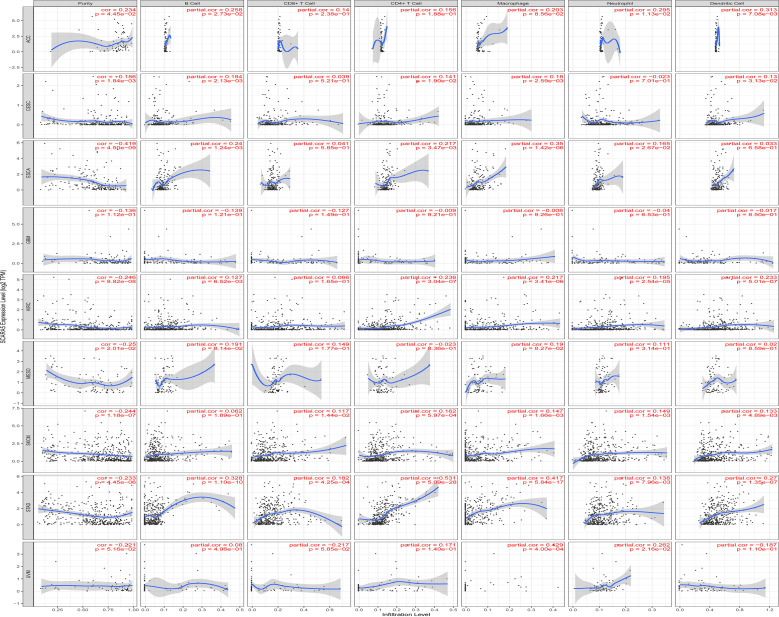
The Tumor IMmune Estimation Resource (TIMER) website https://cistrome.shinyapps.io/timer/) is used to view The Cancer Genome Atlas (TCGA) database for ACC, CESC, ESCA, GBM, KIRC, SKCM, MESO, STAD, and UVM in immune cells and the correlation of tumor purity and scavenger receptor class A member 5 (*SCARA5*) levels.

**Figure 7:**
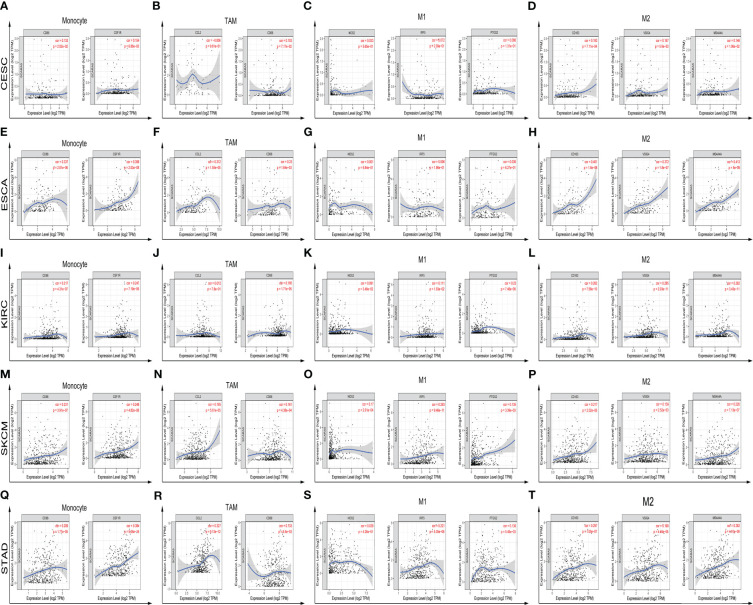
Further analysis of immune infiltration correlations with statistical significance in CESC, ESCA, KIRC, SKCM, STAD, and specific immune molecules (CD86, CSF1R, CCL2, CD68, NOS2, IRF5, PTGS2, CD163, VSIG4, and MS4A4A) **(A–T)**.

The Tumor IMmune Estimation Resource (TIMER) website (https://cistrome.shinyapps.io/timer/) was used to assess the correlation between *SCARA5* levels, tumor purity, and immune infiltration levels in ACC, CESC, ESCA, GBM, KIRC, SKCM, MESO, STAD, and UVM (**Figure 6**). **Table 2** shows the results of the correlation analysis between *SCARA5* expression and immune cell-related genes and biomarkers. In ACC, *SCARA5* expression was positively correlated with purity, B cells, CD8+T cells, neutrophils, and dendritic cells (*p*_s_ < 0.05). In CESC, *SCARA5* expression was positively correlated with B cells, CD4+T cells, macrophages, and dendritic cells (*p*_s_ < 0.05) and negatively correlated with purity (*p* < 0.05). In ESCA, *SCARA5* expression was positively correlated with B cells, CD4+T cells, macrophages, and neutrophils (*p*_s_ < 0.05) and negatively correlated with purity (*p* < 0.05). In GBM, no significant correlations were found between *SCARA5* expression and immune infiltration levels. In contrast, in KIRC, *SCARA5* expression was positively correlated with B cells, CD4+T cells, macrophages, neutrophils, and dendritic cells (*p*_s_ < 0.05) and negatively correlated with purity (*p* < 0.05). In MESO, *SCARA5* expression was positively correlated with purity (*p* < 0.05). In SKCM, *SCARA5* expression was positively correlated with CD8+T cells, CD4+T cells, macrophages, neutrophils, and dendritic cells (*p*_s_ < 0.05) and negatively correlated with purity (*p* < 0.05). In STAD, *SCARA5* expression was positively correlated with B cells, CD8+T cells, CD4+T cells, macrophages, neutrophils, and dendritic cells (*p*_s_ < 0.05). In UVM, *SCARA5* expression was positively correlated with macrophages and neutrophils (*p*_s_ < 0.05). We further found significant correlations with immune infiltration in CESC, ESCA, KIRC, SKCM, STAD, and specific immune molecules (*CD86, CSF1R, CCL2, CD68, NOS2, IRF5, PTGS2, CD163, VSIG4*, and *MS4A4A*) (**Figure 7**). In CESC, *SCARA5* expression was positively correlated with *CD163, VSIG4*, and *MS4A4A* (*p*_s_ < 0.05). In ESCA, *SCARA5* expression was positively correlated with *CD86, CSF1R, CCL2, CD68, CD163, VSIG4*, and *MS4A4A* (*p*_s_ < 0.05). Moreover, in KIRC, *SCARA5* expression was positively correlated with *CD86, CSF1R, CD68, NOS2, IRF5, PTGS2, CD163, VSIG4*, and *MS4A4A* (*p*_s_ < 0.05). In SKCM, *SCARA5* expression was positively correlated with *CD86, CSF1R, CCL2, CD68, NOS2, IRF5, PTGS2, CD163, VSIG4*, and *MS4A4A* (*p*_s_ < 0.05). Finally, in STAD, *SCARA5* expression was positively correlated with *CD86, CSF1R, CCL2, CD68, IRF5, PTGS2, CD163, VSIG4*, and *MS4A4A* (*p*_s_ < 0.05). **Table 3** shows the correlation analysis between *SCARA5* and immune cell-related genes and markers in Gene Expression Profiling Interactive Analysis (GEPIA) (cancer-pku.cn). The results are consistent with previous searches in the TIMER database.

**Table 2:** Correlation analysis between scavenger receptor class A member 5 (*SCARA5*) and immune cell-related genes and markers in Tumor IMmune Estimation Resource (TIMER).

Description	Gne markers	CESC		ESCA		KIRC		SKCM		STAD	
		R	P	R	P	R	P	R	P	R	P
CD8+T cell	CD8A	0.110	0.045	0.270	0.001	0.170	0.000	0.200	0.000	0.250	0.000
	CD8B	0.078	0.173	0.260	0.001	0.180	0.000	0.210	0.000	0.200	0.000
T cell (general)	CD3D	0.098	0.088	0.280	0.000	0.190	0.000	0.230	0.000	0.270	0.000
	CD3E	0.140	0.012	0.320	0.000	0.200	0.000	0.240	0.000	0.320	0.000
	CD2	0.11	0.047	0.290	0.000	0.180	0.000	0.240	0.000	0.280	0.000
B cell	CD19	0.260	0.000	0.420	0.000	0.400	0.000	0.220	0.000	0.530	0.000
	CD79A	0.250	0.000	0.390	0.000	0.35	0.000	0.180	0.000	0.540	0.000
Monocyte	CD86	0.110	0.052	0.350	0.000	0.160	0.000	0.230	0.000	0.160	0.002
	CSF1R	0.160	0.005	0.410	0.000	0.190	0.000	0.240	0.000	0.32	0.000
TAM	CCL2	0.130	0.022	0.350	0.000	0.014	0.739	0.190	0.000	0.330	0.000
	CD68	0.059	0.306	0.091	0.247	0.140	0.001	0.093	0.043	-0.021	0.686
M1 macrophage	NOS2	-0.021	0.709	-0.016	0.838	0.022	0.612	0.130	0.004	0.023	0.657
	IRF5	0.025	0.663	0.11	0.158	0.130	0.003	0.28	0.000	0.160	0.002
	PTGS2	0.061	0.291	-0.01	0.895	0.210	0.000	0.12	0.009	0.077	0.137
M2 macrophage	CD163	0.170	0.002	0.400	0.000	0.180	0.000	0.2	0.000	0.190	0.000
	VSIG4	0.140	0.015	0.400	0.000	0.220	0.000	0.130	0.006	0.140	0.006
	MS4A4A	0.140	0.015	0.43	0.000	0.210	0.000	0.22	0.000	0.220	0.000
Neutrophils	CEACAM8	-0.015	0.791	0.062	0.431	0.13	0.004	0.051	0.27	-0.005	0.928
	ITGAM	0.095	0.099	0.32	0.000	0.210	0.000	0.2	0.000	0.280	0.000
	CCR7	0.280	0.000	0.460	0.000	0.240	0.000	0.290	0.000	0.580	0.000
Natural killer cell	KIR2DL1	-0.011	0.851	0.095	0.229	-0.021	0.626	0.120	0.010	0.100	0.053
	KIR2DL3	0.110	0.047	0.074	0.349	0.052	0.228	0.170	0.000	0.055	0.288
	KIR2DL4	0.021	0.717	0.14	0.085	0.100	0.020	0.13	0.004	-0.034	0.511
	KIR3DL1	0.098	0.089	0.210	0.008	0.004	0.923	0.16	0.001	0.130	0.012
	KIR3DL2	0.120	0.033	0.098	0.213	-0.002	0.973	0.190	0.000	0.140	0.008
	KIR3DL3	0.089	0.122	0.039	0.623	0.085	0.050	0.015	0.751	-0.053	0.308
	KIR2DS4	0.120	0.039	0.049	0.535	-0.016	0.705	0.110	0.013	0.028	0.591
Dendritic cell	HLA-DPB1	0.077	0.181	0.380	0.000	0.094	0.029	0.220	0.000	0.210	0.000
	HLA-DQB1	0.063	0.270	0.26	0.001	0.018	0.671	0.19	0.000	0.150	0.004
	HLA-DRA	0.05	0.381	0.310	0.000	0.087	0.044	0.230	0.000	0.120	0.021
	HLA-DPA1	0.02	0.724	0.31	0.000	0.089	0.04	0.190	0.000	0.160	0.002
	BDCA-1(CD1C)	0.150	0.008	0.540	0.000	0.220	0.000	0.410	0.000	0.640	0.000
	BDCA-4(NRP1)	0.170	0.003	0.370	0.000	-0.034	0.435	0.380	0.000	0.310	0.000
	CD11c (ITGAX)	0.210	0.000	0.350	0.000	0.220	0.000	0.210	0.000	0.22	0.000
Th1	T-bet (TBX21)	0.110	0.059	0.300	0.000	0.100	0.018	0.220	0.000	0.26	0.000
	STAT4	0.160	0.006	0.41	0.000	0.210	0.000	0.260	0.000	0.38	0.000
	STAT1	-0.056	0.331	0.140	0.067	0.13	0.002	0.087	0.059	-0.150	0.004
	IFN-γ (IFNG)	-0.02	0.730	0.120	0.138	0.190	0.000	0.15	0.001	-0.054	0.295
	TNF-α (TNF)	0.009	0.869	0.044	0.576	0.160	0.000	0.19	0.000	0.130	0.011
Th2	GATA3	0.006	0.919	0.280	0.000	0.240	0.000	0.29	0.000	0.260	0.000
	STAT6	-0.008	0.891	-0.046	0.561	0.059	0.172	-0.009	0.844	0.130	0.012
	STAT5A	-0.016	0.774	0.300	0.000	0.220	0.000	-0.023	0.619	0.240	0.000
	IL13	0.082	0.152	0.270	0.000	0.110	0.01	0.083	0.070	0.220	0.000
Tfh	BCL6	-0.017	0.767	0.12	0.144	0.045	0.294	0.190	0	0.320	0
	IL21	0.073	0.203	IL21	0.001	0.150	0.000	0.240	0.000	0.220	0.000
Th17	STAT3	0.057	0.316	0.160	0.048	0.088	0.042	0.200	0.000	0.210	0.000
	IL17A	-0.008	0.885	-0.18	0.022	0.130	0.002	-0.023	0.616	-0.049	0.342
Treg	FOXP3	0.170	0.003	0.340	0.000	0.280	0.000	0.220	0.000	0.220	0
	CCR8	0.19	0.001	0.340	0.000	0.250	0.000	0.270	0.000	0.240	0
	STAT5B	0.200	0.000	0.250	0.001	-0.052	0.228	0.210	0.000	0.430	0.000
	TGFβ (TGFB1)	0.021	0.709	0.075	0.342	0.13	0.002	0.230	0.000	0.28	0.000
T cell exhaustion	PD-1 (PDCD1)	0.051	0.373	0.220	0.005	0.190	0.000	0.170	0.000	0.19	0.000
	CTLA4	0.100	0.078	0.280	0.000	0.19	0.000	0.27	0.000	0.150	0.003
	LAG3	0.012	0.829	0.180	0.020	0.220	0.000	0.11	0.015	0.038	0.466
	TIM-3 (HAVCR2)	0.084	0.143	0.330	0.000	-0.038	0.381	0.210	0.000	0.110	0.035
	GZMB	0.038	0.510	0.140	0.069	0.095	0.027	0.170	0.000	-0.071	0.169

CC, Cellular component; BP, Biological process; MF, Molecular function; KEGG, Kyoto Encyclopedia of Genes and Genomes TAM,Tumor Associated Macrophage; ACC, Adrenocortical carcinoma; BLCA, Bladder Urothelial Carcinoma; CESC, Cervical squamous cell carcinoma and endocervical adenocarcinoma; CHOL, Cholangiocarcinoma; COAD, Colon adenocarcinoma; COADREAD, Colon adenocarcinoma/Rectum adenocarcinoma Esophageal carcinoma; DLBC, Lymphoid Neoplasm Diffuse Large B-cell Lymphoma; ESCA, Esophageal carcinoma; GBM ,Glioblastoma multiforme; GBMLGG, Glioma; HNSC, Head and Neck squamous cell carcinoma; KICH, Kidney Chromophobe; KIRC, Kidney renal clear cell carcinoma; KIRP, Kidney renal papillary cell carcinoma; LAML, Acute Myeloid Leukemia; LGG, Brain Lower Grade Glioma; LIHC, Liver hepatocellular carcinoma; LUAD, Lung adenocarcinoma; LUSC, Lung squamous cell carcinoma; MESO, Mesothelioma; OV, Ovarian serous cystadenocarcinoma; PAAD, Pancreatic adenocarcinoma; PCPG, Pheochromocytoma and Paraganglioma; PRAD, Prostate adenocarcinoma; READ, Rectum adenocarcinoma; SARC, Sarcoma; SKCM, Skin Cutaneous Melanoma; STAD, Stomach adenocarcinoma; STES, Stomach and Esophageal carcinoma; TGCT, Testicular Germ Cell Tumors; THCA, Thyroid carcinoma; THYM, Thymoma; UCEC, Uterine Corpus Endometrial Carcinoma; UCS, Uterine Carcinosarcoma; UVM, Uveal Melanoma.

**Table 3:** Correlation analysis between scavenger receptor class A member 5 (*SCARA5*) and immune cell-related genes and markers in Gene Expression Profiling Interactive Analysis (GEPIA).

Description	Gene markers	CESC		ESCA		KIRC		SKCM		STAD	
		R	P	R	P	R	P	R	P	R	P
Monocyte	CD86	0.12	0.032	0.34	3.1e−06	0.21	1.3e−06	0.24	1.7e−07	0.19	0.00016
	CSF1R	0.16	0.0054	0.39	4.8e−08	0.25	9.9e−09	0.26	2.5e−08	0.35	3.5e−13
TAM	CCL2	0.13	0.022	0.32	1.3e−05	0.017	0.71	0.2	1.8e−05	0.31	1e−10
	CD68	0.11	0.061	0.22	0.0035	0.18	4.6e−05	0.19	5.7e−05	0.096	0.052
M1 macrophage	NOS2	0.0075	0.9	−0.0058	0.94	0.096	0.027	0.17	3e−04	0.042	0.4
	IRF5	0.056	0.33	0.093	0.21	0.12	0.0069	0.3	9.6e−11	0.18	0.00034
	PTGS2	0.1	0.078	0.026	0.72	0.24	5.3e−08	0.14	0.002	0.16	0.00086
M2 macrophage	CD163	0.14	0.013	0.4	2.2e−08	0.29	1.7e−11	0.2	1.3e−05	0.15	0.0026
	VSIG4	0.14	0.016	0.37	1.9e−07	0.28	4.3e−11	0.14	0.0037	0.15	0.0021
	MS4A4A	0.14	0.018	0.43	1.7e−09	0.27	3.4e−10	0.22	1.3e−06	0.25	4.1e−07

CC, Cellular component; BP, Biological process; MF, Molecular function; KEGG, Kyoto Encyclopedia of Genes and Genomes TAM,Tumor Associated Macrophage; ACC, Adrenocortical carcinoma; BLCA, Bladder Urothelial Carcinoma; CESC, Cervical squamous cell carcinoma and endocervical adenocarcinoma; CHOL, Cholangiocarcinoma; COAD, Colon adenocarcinoma; COADREAD, Colon adenocarcinoma/Rectum adenocarcinoma Esophageal carcinoma; DLBC, Lymphoid Neoplasm Diffuse Large B-cell Lymphoma; ESCA, Esophageal carcinoma; GBM ,Glioblastoma multiforme; GBMLGG, Glioma; HNSC, Head and Neck squamous cell carcinoma; KICH, Kidney Chromophobe; KIRC, Kidney renal clear cell carcinoma; KIRP, Kidney renal papillary cell carcinoma; LAML, Acute Myeloid Leukemia; LGG, Brain Lower Grade Glioma; LIHC, Liver hepatocellular carcinoma; LUAD, Lung adenocarcinoma; LUSC, Lung squamous cell carcinoma; MESO, Mesothelioma; OV, Ovarian serous cystadenocarcinoma; PAAD, Pancreatic adenocarcinoma; PCPG, Pheochromocytoma and Paraganglioma; PRAD, Prostate adenocarcinoma; READ, Rectum adenocarcinoma; SARC, Sarcoma; SKCM, Skin Cutaneous Melanoma; STAD, Stomach adenocarcinoma; STES, Stomach and Esophageal carcinoma; TGCT, Testicular Germ Cell Tumors; THCA, Thyroid carcinoma; THYM, Thymoma; UCEC, Uterine Corpus Endometrial Carcinoma; UCS, Uterine Carcinosarcoma; UVM, Uveal Melanoma.

### 3.7 Gene sets enriched in *SCARA5* expression phenotype and protein–protein interaction in SKCM

**Figure 8:**
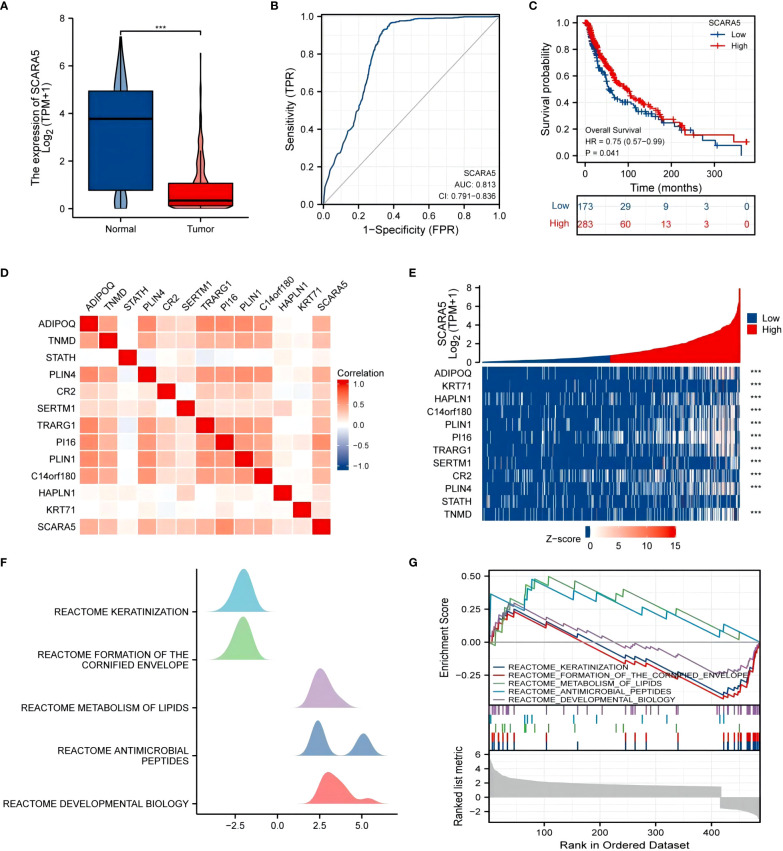
UCSC XENA (https://xenabrowser.net/datapages/) unified RNA sequencing (RNAseq) data of The Cancer Genome Atlas (TCGA) and Genotype-Tissue Expression (GTEx) in transcripts per million reads (TPM) showed that scavenger receptor class A member 5 (*SCARA5*) levels in tumor tissue were lower than those in normal tissue, and the difference was statistically significant (***p < 0.001), using “ggplot2” for visualization **(A)**. The “pROC” package was used to analyze the diagnostic value of *SCARA5* in the SKCM tumor group and the normal group, and the “ggplot2” package was used to draw the receiver operating characteristic (ROC) curve **(B)**. The difference in survival time distribution in SKCM was statistically significant (*p* = 0.04) **(C)**. The “DESeq2” package was used to analyze the RNAseq data in the level 3 High-throughout sequencing Counts (HTSeq-Counts) format in TCGA (https://portal.gdc.cancer.gov/) SKCM and select molecules (*KRT71, HAPLN1, C14orf180, PLIN1, PI16, TRARG1, SERTM1, CR2, PLIN4, STATH, TNMD*, and *ADIPOQ*) with higher differences with *SCARA5* used to draw a correlation heatmap **(D)** and single-gene co-expression heatmap **(E)**. The gene set enrichment analysis (GSEA) mountain map **(F)** and GSEA enrichment map **(G)**.

**Figure 9:**
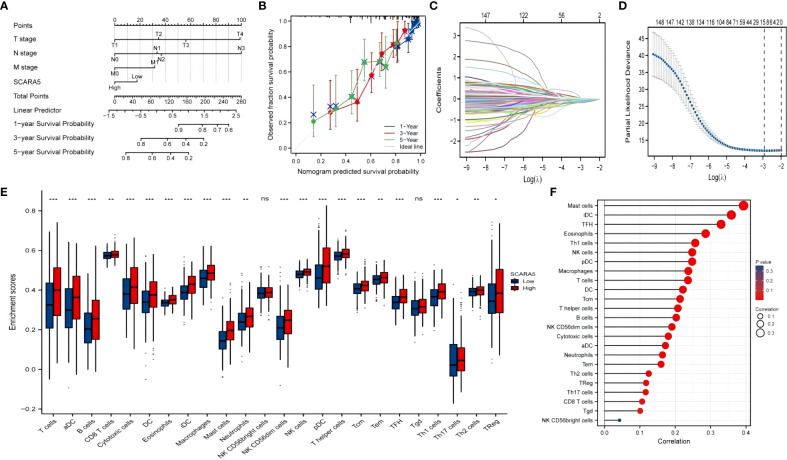
Bioinformatics were used to analyze the prognosis of scavenger receptor class A member 5 (*SCARA5*) and its correlation with immune infiltration. The “rms” package and the “survival” package were used to predict the prognosis of *SCARA5* at 1, 3, and 5 years after the onset of SKCM **(A)**. **(B)** shows calibration visualization. The abscissa is the survival probability predicted by the model, the ordinate is the actual observed survival probability, and the gray diagonal line is the ideal line. The lines and points of different colors (except for the gray diagonal line) represent the predictions of the model at different time points. When the lines of different colors are closer to the ideal gray line, and the error bar is smaller (stable), this indicates that at this time point the prediction effect is better. Differential genes in the GSE100050 data set were selected and the “glmnet” package used to draw a Lasso coefficient screening diagram and simultaneously plot the Lasso variable trajectory **(C, D)**. Gene Set Variation Analysis (GSVA) package and immune infiltration algorithm ssGSEA (GSVA package built-in algorithm) were used to map *SCARA5* and SKCM with 24 kinds of immune cells [aDC (activated DC); B cells; CD8 T cells; cytotoxic cells; DC; eosinophils; iDC (immature DC); macrophages; mast cells; neutrophils; NK CD56bright cells; NK CD56dim cells; NK cells; pDC (Plasmacytoid DC); T cells; T helper cells; Tcm (T central memory); Tem (T effector memory); Tfh (T follicular helper); Tgd (T gamma delta); Th1 cells; Th17 cells; Th2 cells; Treg] [*P<0.05, **P<0.01, ***P<0.001, and ns (no significant)] **(E, F)**.

University of California Santa Cruz Xena (UCSC XENA) Tools is an R package for accessing genomics data from the UCSC Xena platform, from cancer multi-omics to single-cell RNA-seq (33). GTEx data from TCGA and the corresponding normal tissue data in SKCM (skin melanoma) were extracted for the present study. The Wilcoxon rank-sum test showed that the expression of *SCARA5* was significantly lower in tumors than in normal subjects (*p* < 0.001) and the results were visualized using “ggplot2” (**Figure 8A**). The “pROC” package was used to analyze the diagnostic value of *SCARA5* in the SKCM tumor group and the normal group, and the “ggplot2” package was used to draw the receiver operating characteristic (ROC) curve. The abscissa is the false positive rate (FPR) and the ordinate is the true positive rate (TPR). *SCARA5* expression exhibited good diagnostic performance in differentiating tumor and normal tissues (**Figure 8B**). The difference in survival time in SKCM was statistically significant (*p* = 0.04, **Figure 8C**). The R package “DESeq2” (34) was used to analyze the RNAseq data in the High-throughout sequencing Counts (HTSeq-Counts) format in TCGA (https://portal.gdc.cancer.gov/) SKCM, and the molecules *KRT71, HAPLN1, C14orf180, PLIN1, PI16, TRARG1, SERTM1, CR2, PLIN4, STATH, TNMD*, and *ADIPOQ*, with higher differences in *SCARA5* expression, selected to draw a correlation heatmap (**Figure 8D**) and single-gene co-expression heatmap (**Figure 8E**). The GSEA enrichment plots (**Figures 8F, G**) of the 12 molecules, including *SCARA5*, were analyzed, and significant enrichment in Reactome developmental biology, Reactome antimicrobial peptides, Reactome metabolism of lipids, Reactome formation of the cornified envelope, and Reactome keratinization was observed. Based on multiple regression analyses, the scale score was set to represent each variable in the multiple regression model, and the probability of event occurrence was predicted by calculating the final total score (35). The “rms” package and the “survival” package were used to predict the prognostic value of *SCARA5* at 1, 3, and 5 years after the onset of SKCM (**Figure 9A**). **Figure 9B** shows the calibration curve. The abscissa is the survival probability predicted by the model, the ordinate is the actual survival probability, and the gray diagonal line is the ideal line. The differential genes in the GSE100050 data set were used to generate the Lasso coefficient profile and plot the Lasso variable trajectory (**Figures 9C, D**). The Gene Set Variation Analysis (GSVA) package and the immune infiltration algorithm ssGSEA (GSVA package built-in algorithm) were used to map *SCARA5* and SKCM with 24 kinds of immune cells [aDC (activated DC); B cells; CD8 T cells; cytotoxic cells; DC; eosinophils; iDC (immature DC); macrophages; mast cells; neutrophils; NK CD56 bright cells; NK CD56 dim cells; NK cells; pDC (plasmacytoid DC); T cells; T helper cells; Tcm (T central memory); Tem (T effector memory); Tfh (T follicular helper); Tgd (T gamma delta); Th1 cells; Th17 cells; Th2 cells; and Treg] (**Figures 9E, F**) (36, 37).

### 3.8 Correlation of *SCARA5* expression with clinicopathological features and outcomes in tissue samples

**Figure 10:**
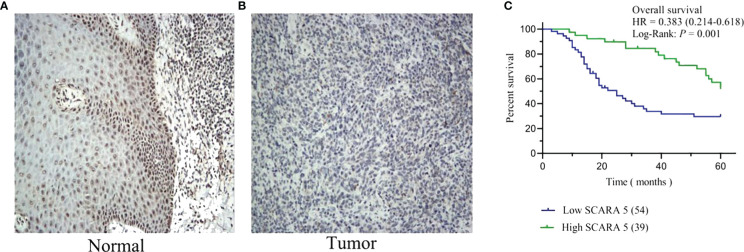
Expression of scavenger receptor class A member 5 (*SCARA5*) in SKCM and survival analysis of our cases. High *SCARA5* expression (IRS 4–12) **(A)** and low expression (IRS 0–3) **(B)**. Through Kaplan–Meier curve assessment, patients with low *SCARA5* expression were shown to have a significantly lower 5-year survival rate than those with high *SCARA5* expression (**(C)** *p* = 0.001, log-rank test).

**Figure 11:**
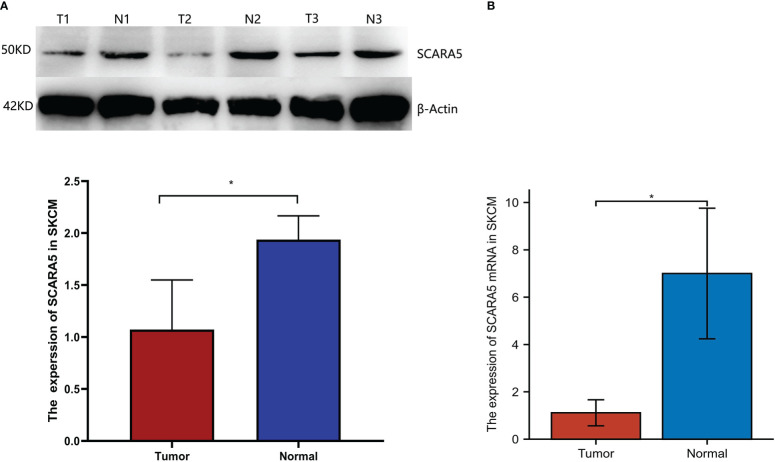
Expression of scavenger receptor class A member 5 (*SCARA5*) in SKCM. Western blotting showing expression of *SCARA5* in three SKCM tissues and adjacent normal tissues. Glyceraldehyde-3-Phosphate Dehydrogenase (GADPH) added as a loading control **(A)**. *p < 0.05. Reverse transcription PCR detection of *SCARA5* mRNA expression in three SKCM tissues and adjacent normal tissues **(B)**. N, normal tissue; T, tumor.

We further investigated whether *SCARA5* affects the progression of SKCM by studying the correlation between *SCARA5* expression and clinicopathological characteristics in our sample tissues. According to the Immunohistochemistry (IHC) results of *SCARA5* expression, SKCM patients were divided into high expression (IRS 4–12) (**Figure 10A**) and low expression (IRS 0–3) (**Figure 10B**) groups. High *SCARA5* expression was detected in 39/93 tumor tissues (41.9%) and low *SCARA5* expression in 54/93 tumor samples (58%). The association between *SCARA5* expression and clinicopathological findings in our samples is shown in **Table 2**. High *SCARA5* expression levels were associated with tumor, node, and metastasis (TNM) stage (T: *p* = 0.033, N: *p* = 0.029, M: *p* = 0.036), and metastasis/recurrence (*p* = 0.03), while low *SCARA5* expression was associated with patient age, sex and tumor location. There was no significant correlation between *SCARA5* expression and tumor size. These results suggest that *SCARA5* is lowly expressed in SKCM and *SCARA5* has tumor-suppressive functions in SKCM (**Table 4**). To further verify the results of the previous analysis, we verified the RNA and protein expression of *SCARA5* in SKCM using Western blotting and reverse transcription PCR (**Figures 11A, B**), which showed that *SCARA5* expression levels are significantly reduced in SKCM tissues compared with matched normal tissues.

**Table 4:** Association of scavenger receptor class A member 5 (*SCARA5*) expression and clinicopathological features in melanoma.

Clinicopathological features	*n*	*SCARA5* High expression (*n* = 39) Low expression (*n* = 54)		*p-*value^#^
Sex				0.135
Male	56	20	36	
Female	37	19	18	
Age (years)				0.906
< 60	28	12	16	
≥ 60	65	27	38	
T stage				0.033*
T1	23	14	9	
T2	25	13	12	
T3	31	8	23	
T4	14	4	10	
N stage				0.029*
N0	31	18	13	
N1	27	13	14	
N2	21	5	16	
N3	14	3	11	
M stage				0.036*
M0	80	37	43	
M1	13	2	11	
Tumor tissue site				0.629
Head and neck	16	5	11	
Trunk	22	10	12	
Extremities	55	24	31	
Recurrence				0.030*
No	66	32	33	
Yes	27	7	21	

^#^Chi-squared test; *p < 0.05.

CC, Cellular component; BP, Biological process; MF, Molecular function; KEGG, Kyoto Encyclopedia of Genes and Genomes TAM,Tumor Associated Macrophage; ACC, Adrenocortical carcinoma; BLCA, Bladder Urothelial Carcinoma; CESC, Cervical squamous cell carcinoma and endocervical adenocarcinoma; CHOL, Cholangiocarcinoma; COAD, Colon adenocarcinoma; COADREAD, Colon adenocarcinoma/Rectum adenocarcinoma Esophageal carcinoma; DLBC, Lymphoid Neoplasm Diffuse Large B-cell Lymphoma; ESCA, Esophageal carcinoma; GBM ,Glioblastoma multiforme; GBMLGG, Glioma; HNSC, Head and Neck squamous cell carcinoma; KICH, Kidney Chromophobe; KIRC, Kidney renal clear cell carcinoma; KIRP, Kidney renal papillary cell carcinoma; LAML, Acute Myeloid Leukemia; LGG, Brain Lower Grade Glioma; LIHC, Liver hepatocellular carcinoma; LUAD, Lung adenocarcinoma; LUSC, Lung squamous cell carcinoma; MESO, Mesothelioma; OV, Ovarian serous cystadenocarcinoma; PAAD, Pancreatic adenocarcinoma; PCPG, Pheochromocytoma and Paraganglioma; PRAD, Prostate adenocarcinoma; READ, Rectum adenocarcinoma; SARC, Sarcoma; SKCM, Skin Cutaneous Melanoma; STAD, Stomach adenocarcinoma; STES, Stomach and Esophageal carcinoma; TGCT, Testicular Germ Cell Tumors; THCA, Thyroid carcinoma; THYM, Thymoma; UCEC, Uterine Corpus Endometrial Carcinoma; UCS, Uterine Carcinosarcoma; UVM, Uveal Melanoma.

### 3.9 Correlation between *SCARA5* expression and patient survival

Kaplan–Meier analysis was conducted to assess the difference in overall survival (OS) in SKCM patients between patients with high and low *SCARA5* expression. The results showed that the 5-year survival rate of patients with low *SCARA5* expression was significantly lower than that of patients with high *SCARA5* expression (**Figure 10C**, log-rank test *p* = 0.001).

### 3.10 Univariate and multivariate analyses of prognostic variables in SKCM patients

We performed a univariate analysis of each variable according to the OS of SKCM patients to investigate variables with potential prognostic significance. Differences in prognosis were assessed by determining each variable’s hazard ratio (HR) and *p*-value. The relative importance of each variable was then examined using a Cox proportional hazards model. Multiple stepwise regression analysis confirmed that *SCARA5* expression and histological stage were significant prognostic factors for OS in SKCM patients. Finally, multivariate analysis showed that *SCARA5* protein expression was significantly associated with poor prognosis in SKCM patients and was an independent prognostic factor (**Table 5**).

**Table 5:** Univariate and multivariate analysis of prognostic factors of 5-year overall survival in scavenger receptor class A member 5 (*SCARA5*) patients.

Characteristic	Univariate analysis			Multivariate analysis		
	HR	95%CI	*p*	HR	95%CI	*p*
*SCARA5* expression						
Low vs. high	0.383	0.214–0.687	0.001*	0.433	0.226–0.831	0.012*
Sex						
Male vs. female	1.563	0.884–2.763	0.124			
Age (years)						
< 60 vs. ≥ 60	1.194	0.657–2.171	0.561			
Tumor tissue site						
Trunk Head and neck vs. Extremities	0.981	0.687–1.402	0.918			
T stage						
T1–2 vs. T3–4	1.785	1.360–2.343	<0.001**	2.098	1.134–3.882	0.018*
N stage						
N0 vs. N1–3	3.172	2.354–4.275	<0.001**	3.517	1.519–8.145	0.003*
M stage						
M0 vs. M1	4.893	2.538–9.435	< 0.001**	2.369	1.151–2.060	0.019*
Recurrence						
No vs. yes	4.025	2.309–7.017	< 0.001**	2.021	1.085–3.764	0.027*

CC, Cellular component; BP, Biological process; MF, Molecular function; KEGG, Kyoto Encyclopedia of Genes and Genomes TAM,Tumor Associated Macrophage; ACC, Adrenocortical carcinoma; BLCA, Bladder Urothelial Carcinoma; CESC, Cervical squamous cell carcinoma and endocervical adenocarcinoma; CHOL, Cholangiocarcinoma; COAD, Colon adenocarcinoma; COADREAD, Colon adenocarcinoma/Rectum adenocarcinoma Esophageal carcinoma; DLBC, Lymphoid Neoplasm Diffuse Large B-cell Lymphoma; ESCA, Esophageal carcinoma; GBM ,Glioblastoma multiforme; GBMLGG, Glioma; HNSC, Head and Neck squamous cell carcinoma; KICH, Kidney Chromophobe; KIRC, Kidney renal clear cell carcinoma; KIRP, Kidney renal papillary cell carcinoma; LAML, Acute Myeloid Leukemia; LGG, Brain Lower Grade Glioma; LIHC, Liver hepatocellular carcinoma; LUAD, Lung adenocarcinoma; LUSC, Lung squamous cell carcinoma; MESO, Mesothelioma; OV, Ovarian serous cystadenocarcinoma; PAAD, Pancreatic adenocarcinoma; PCPG, Pheochromocytoma and Paraganglioma; PRAD, Prostate adenocarcinoma; READ, Rectum adenocarcinoma; SARC, Sarcoma; SKCM, Skin Cutaneous Melanoma; STAD, Stomach adenocarcinoma; STES, Stomach and Esophageal carcinoma; TGCT, Testicular Germ Cell Tumors; THCA, Thyroid carcinoma; THYM, Thymoma; UCEC, Uterine Corpus Endometrial Carcinoma; UCS, Uterine Carcinosarcoma; UVM, Uveal Melanoma; HR, hazard ratio; CI, confidence interval; *p < 0.05, **p < 0.01.

## 4 Discussion

Malignant melanoma is one of the malignancies with the highest metastatic potential and is the most lethal skin cancer worldwide (38). The high mortality of malignant melanoma is associated with the occurrence of melanoma metastases. Metastatic melanoma is very aggressive and resistant to currently available chemotherapy and immunotherapy (39). Given that T-cell infiltration is often found in malignant melanoma tumors, inhibition of immune checkpoints is a potential therapeutic modality (40). Identifying novel epitopes from oncogenic mutations, such as tumor vaccines and adoptively transferred tumor-reactive T cells, is important for improving immunotherapy’s efficacy. Moreover, checkpoint blockade therapy in immunotherapy can stimulate cytotoxic T lymphocytes to recognize these neo-epitopes in patients (41).

*SCARA5* is a member of the scavenger receptor family located on chromosome 8p21 (a region frequently deleted in human cancers). *SCARA5* has been shown to act as a tumor suppressor gene to suppress various cancers (42). For instance, Yan et al. found that *SCARA5* significantly inhibited gastric cancer cells, with an inhibitory effect of 69.4%, tumor proliferation index of 23.3%, apoptotic index of 47.3%, and reduced tumor angiogenesis (43). Huang et al. documented methylation in the *SCARA5* promoter region of Hepatocellular carcinoma (HCC) cells, accounting for low *SCARA5* expression and hence enhanced *in vivo* tumorigenicity, cell invasion, and tumor metastasis. In contrast, *SCARA5* overexpression inhibits tumorigenicity, cell invasion, and metastasis (11). In addition, Zhang et al. showed that *SCARA5* inhibits the invasive function of gastric cancer cells by affecting the initiation of Epithelial-mesenchymal transition (EMT) (14). However, its role in skin malignant melanoma remains unclear.

In human organs, *SCARA5* is mainly found in the airways, developing aorta, and muscle bundles, and is abundantly expressed in gonadal epithelial cells (10). Interestingly, *SCARA5* acts as a ferritin receptor to mediate non-transferrin iron transmission (44). Extracellular ferritin, a non-transferrin siderophore that various cells can endocytose, was shown by *in situ* hybridization to be specifically located at the cell edge. Significant upregulation of *SCARA5* was observed, while ferritin uptake was observed in both embryos (44). Together, these findings indicate that *SCARA5* has a relatively broad tissue distribution and can internalize ferritin to remove ferritin or transport iron. There is a rich body of literature available suggesting that the degradation of ferritin by autophagy can promote ferroptosis (45–48). Therefore, *SCARA5* with ferritin recognition and uptake functions may be involved in regulating ferritin homeostasis and cell death, suggesting that *SCARA5* may be a potential target for therapeutic strategies in cancer and other diseases (49). Ferroptosis is closely related to immune infiltration, and its damage-associated molecular patterns (DAMPs) can release proinflammatory mediators, such as *HMGB1*. Ferroptosis has recently been associated with T cell-mediated antitumor immunity and the efficacy of tumor immunotherapy. Importantly, ferroptosis contributes to the antitumor effect of CD8+ T cells and determines the efficacy of *anti-PD-1/PD-L1* immunotherapy. It is widely thought that immunotherapy combined with ferroptosis-promoting modalities, such as radiation therapy and targeted therapy, can have a synergistic effect through ferroptosis of promoting tumor control. According to the previous literature, *SCARA5* is highly correlated with immune infiltration, suggesting that *SCARA5* has great potential as a target for ferroptosis when combined with immunotherapy, which has important biological and clinical significance.

In this study, we conducted bioinformatics analysis on high-throughput RNA sequencing data from TCGA to show that *SCARA5* exhibited significantly lower expression in SKCM tissues than in paired normal tissues. Moreover, *SCARA5* can play an inhibitory role in the occurrence and progression of SKCM. ROC analysis yielded an AUC of 0.813, suggesting that *SCARA5* may be a potential diagnostic biomarker of SKCM. Subsequently, we further studied the relationship between *SCARA5*, immune cells, and immune molecules, and found that *SCARA5* is highly correlated with immunity. A review of the literature yielded few studies on the relationship between *SCARA5* and SKCM, and its prognostic value. We constructed a prognostic gene signature model based on the *SCARA5* Kaplan–Meier curve, which yielded a good performance for SKCM survival prediction. We found that the OS, Pulmonary Functional Imaging (PFI), and Disease-Specific Survival (DSS) were poorer in SKCM patients in the low-*SCARA5*-expression group. Multivariate analysis showed that *SCARA5* is an independent factor affecting the survival of SKCM patients (*p* < 0.05) and can be used as a biomarker of SKCM.

We found that *SCARA5* expression in malignant melanoma was significantly correlated with immune infiltration levels. Overwhelming evidence indicates that the malignant melanoma microenvironment contributes to the immunological changes during SKCM progression, suggesting that *SCARA5* may play an important role in the immune system. Therefore, this study compared the differences in immune cell infiltration between patients with high and low expression of *SCARA5*. In recent years, much emphasis has been placed on better understanding the tumor microenvironment. It is widely thought that the tumor microenvironment can promote the occurrence, development, recurrence, and metastasis of tumors and is an important structure in the body (50, 51). A comprehensive analysis of tumor-infiltrating cells, cytokines/chemokines, gene expression, etc. to formulate individualized and precise immunotherapy for patients with malignant melanoma is of great significance for effectively evaluating and predicting the efficacy of immunotherapy.

Moreover, we demonstrated the clinical prognostic value of *SCARA5*. It has been shown that the expression of *SCARA5* is related to malignant melanoma, with a significant correlation with TNM stage and recurrence. Based on TCGA database analysis, our prognostic model showed that *SCARA5* expression has a high prognostic value. Based on our integrated analysis of the GEO and TCGA databases, we believe that *SCARA5* can be used as an effective prognostic indicator, playing an important role in guiding the individualized treatment of SKCM patients.

Some limitations in the present study should be acknowledged. First, we only preliminarily investigated the role of *SCARA5* expression in SKCM. Further validation through *in vitro* and *in vivo* experiments is warranted to investigate the underlying molecular mechanisms and their biological functions to deepen our understanding of the direct effects of *SCARA5* on SKCM. Indeed, more clinical information on tumor progression and prognosis is warranted to better understand the relationship between *SCARA5* and SKCM.

## 5 Conclusion

In this study, we found that low expression of *SCARA5* was significantly associated with poor prognosis in SKCM patients and could promote the progression of SKCM, suggesting its value as a potential biomarker for SKCM. Through immune infiltration analysis and GSEA, we demonstrated that *SCARA5* also plays a very important role in the tumor immune microenvironment, providing a foothold for future studies on precise and individualized treatment of malignant melanoma. Finally, more population-based studies with larger sample sizes and functional studies are required to confirm our findings.

## Data availability statement

The original contributions presented in the study are included in the article/**Supplementary Material**. Further inquiries can be directed to the corresponding author.

## Ethics statement

The studies involving human participants were reviewed and approved by the Affiliated Hospital of Nantong University. The patients/participants provided their written informed consent to participate in this study.

## Author contributions

ZG contributed to the conception and design of the review. QN and XL wrote the manuscript. HH collected clinical patient data and completed immunohistochemical experiments. QN and XL were co-first authors. All authors contributed to the article and approved the submitted version.
